# Antithrombotic management after aortic valve replacement with biological prosthesis: a meta-analysis

**DOI:** 10.1186/s13019-024-02863-z

**Published:** 2024-06-26

**Authors:** Mikko Uimonen, Ilari Kuitunen, Ville Ponkilainen, Ari Mennander, Mikko S. Mattila

**Affiliations:** 1 Heart Hospital, Tampere University Hospital, Wellbeing Services County of Pirkanmaa, Elämänaukio 1, 33520 Tampere, Finland; 2 Faculty of Medicine and Health Technology, Tampere University, Tampere, Finland; 3 Institute of Clinical Medicine, University of Eastern Finland, Kuopio, Finland; 4 Department of Pediatrics, Kuopio University Hospital, Kuopio, Finland; 5 Department of Orthopedics, Tampere University Hospital, Wellbeing Services County of Pirkanmaa, Tampere, Finland

**Keywords:** Antithrombotic medication, bioAVR, Biological aortic valve replacement

## Abstract

**Background:**

We aimed to summarise the existing knowledge regarding antithrombotic medications following surgical aortic valve replacement (SAVR) using a biological valve prosthesis.

**Methods:**

We performed a meta-analysis of studies that reported the results of using antithrombotic medication to prevent thromboembolic events after SAVR using a biological aortic valve prosthesis and recorded the outcomes 12 months after surgery. Since no randomised controlled trials were identified, observational studies were included. The analyses were conducted separately for periods of 0–12 months and 3–12 months after surgery. A random effects model was used to calculate pooled outcome event rates and 95% confidence intervals (CIs).

**Results:**

The search yielded eight eligible observational studies covering 6727 patients overall. The lowest 0- to 12-month mortality was observed in patients with anticoagulation (2.0%, 95% CI 0.4–9.7%) and anticoagulation combined with antiplatelet therapy (2.2%, 95% CI 0.9–5.5%), and the highest was in patients without antithrombotic medication (7.3%, 95% CI 3.6–14.2%). Three months after surgery, mortality was lower in anticoagulant patients (0.5%, 95% CI 0.1–2.6%) than in antiplatelet patients (3.0%, 95% CI 1.2–7.4%) and those without antithrombotics (3.5%, 95% CI 1.3–9.3%). There was no eligible evidence of differences in stroke rates observed among medication strategies. At 0- to 12-month follow-up, all antithrombotic treatment regimens resulted in an increased bleeding rate (antiplatelet 4.2%, 95% CI 2.9–6.1%; anticoagulation 7.5%, 95% CI 3.8–14.4%; anticoagulation combined with antiplatelet therapy 8.3%, 95% CI 5.7–11.8%) compared to no antithrombotic medication (1.1%, 95% CI 0.4–3.4%). At 3- to 12-month follow-up, there was up to an eight-fold increase in the bleeding rate in patients with anticoagulation combined with antiplatelet therapy when compared to those with no antithrombotic medication. Overall, the evidence certainty was ranked as very low.

**Conclusion:**

Although this meta-analysis reveals that anticoagulation therapy has a beneficial tendency in terms of mortality at 1 year after biological SAVR and suggests potential advantages in continuing anticoagulation beyond 3 months, it is limited by very low evidence certainty. The imperative for cautious interpretation and the urgent need for more robust randomised research underscore the complexity of determining optimal antithrombotic strategies in this patient population.

**Supplementary Information:**

The online version contains supplementary material available at 10.1186/s13019-024-02863-z.

## Introduction

The prevention of stroke and thrombosis following aortic valve bioprosthetic implantation is a critical aspect of patient care that necessitates a careful balance of anticoagulants and antiplatelet agents. While lifelong warfarin therapy is recommended for mechanical valves, the approach for bioprosthetic valves is nuanced. The months following initial surgery carry an elevated risk of blood clot formation due to surgery-induced inflammation and tissue healing [1–5]. During this period, the body’s own endothelial cells gradually adhere to the bioprosthetic valve’s surface, offering protection against blood clot formation [6, 7].

Both the American Heart Association (AHA) and the European Society of Cardiology (ESC) currently recommend a 3-month course of warfarin post-bioprosthetic implantation [8, 9]. Intriguingly, previous studies have suggested the potential efficacy of aspirin (ASA) or even treatment completely without antithrombotic medications as comparable alternatives to warfarin in the initial 3 months for emboli and stroke prevention [10, 11]. However, there remains a scarcity of research beyond this initial period, leaving uncertainties regarding optimal anticoagulation and antiplatelet strategies.

The divergent recommendations between AHA and ESC complicate the decision-making process. While AHA advocates the continued use of ASA, ESC, in its latest guidelines, has retracted its prior endorsement of ASA as a permanent solution after aortic bioprosthetic implantation [8, 9]. This discrepancy underscores the complexity of therapeutic decisions, especially considering the intersection of valve disease management with coexisting conditions, such as atrial fibrillation, warranting anticoagulation, or cardio- and cerebrovascular diseases, where antiplatelet therapy may be preferable.

Considering these complexities, our aim is to provide a comprehensive summary of the existing knowledge regarding antithrombotic medications after the initial 3 months following surgical aortic valve replacement (SAVR) using a biological valve prosthesis. We hypothesise that a more extensive antithrombotic medication regimen would be related to lower mortality and stroke rates at the cost of higher bleeding rates.

## Methods

This meta-analysis was conducted according to the guidelines in the Cochrane Handbook and reported according to the Preferred Reporting Items for Systematic Reviews and Meta-Analyses (PRISMA) guidelines and the Meta-analysis of Observational Studies in Epidemiology (MOOSE) checklist [12–14].

### Search and screening process

The search for this systematic review was performed on 19 January 2024. PubMed, Scopus and Web of Science databases were searched from their inception. The search strategy was as follows: ‘biological AND “aortic valve” AND (replacement OR implantation) AND (antithrombotic OR antiplatelet OR anticoagulation)’. We did not use any filters. Abstract and full-report screening were performed independently by two authors (MU and IK). Covidence software was used in the screening process. We did not search grey literature. We searched the reference lists of the included studies by hand to find any missed relevant studies for inclusion.

### Inclusion and exclusion criteria

In the initial screening, we included randomised controlled trials (RCTs) that compared medical treatment regimens to prevent thromboembolic events after SAVR using biological aortic valve prosthesis and reported outcomes 12 months after surgery. In the original protocol, we stated that if there were no eligible RCTs, we would include observational studies (both prospective and retrospective cohort and case-control studies). Studies that did not report original data were excluded. Studies were not excluded based on language restrictions. Transcatheter aortic valve implantation (TAVI) procedures were excluded. Concomitant coronary artery bypass grafting surgery was not regarded as a criterion for exclusion. Paediatric patients, pregnant patients and patients with renal insufficiency requiring dialysis were excluded.

### Patients

Patients were required to have undergone SAVR using a biological aortic valve prosthesis. Both chronic and acute phase surgery patients were included. SAVR was defined as a procedure in which the diseased native aortic valve is removed and replaced by a biological valve prosthesis in an open-heart surgery operation.

### Intervention

We included all medical treatment regimens targeted against thromboembolic events, including agents affecting blood coagulation and thrombus formation. After the screening, we classified medication strategies into the following groups: 1) antiplatelets, including aspirin and P2Y_12_ inhibitors (clopidogrel, ticagrelor); 2) anticoagulation (vitamin K antagonists, such as warfarin; direct factor Xa inhibitors, such as rivaroxaban, apixaban and edoxaban); 3) a combination of anticoagulation and antiplatelet agents; 4) 3-month anticoagulation postoperatively and antiplatelet thereafter; and 5) no medication against thromboembolic events. In all medication strategies, the treatment was initiated immediately after surgery and continued for at least 1 year postoperatively.

### Outcomes

Our main outcome was mortality. The follow-up time was set at 12 months. Secondary outcomes were strokes (haemorrhagic and ischemic, combined) and bleeding complications during the 12-month follow-up period. We used the definitions used by the original studies for all outcomes.

### Data extraction

One author extracted the data and another author validated the extracted data to reduce possible errors. The following information was extracted from each study: authors, inclusion and exclusion criteria, study period, country, intervention definition, control definition, outcome definitions, number of included patients, number of events and main outcome measures.

### Evidence certainty

Evidence certainty was assessed according to the Grading of Recommendations Assessment, Development and Evaluation (GRADE) framework [15]. Evidence certainty was ranked from very low to high. Within-study bias was assessed according to the Risk of Bias in Non-randomized Studies – of Interventions (ROBINS-I, 2016) assessment tool [16, 17]. Two authors (MU and IK) independently conducted the ROBINS-I assessments, and disagreements were resolved by reaching a mutual consensus. The risk of bias figure was created using the Robvis shiny app [18].

### Statistical methods

Statistical analysis was performed using R statistical software (version 4.3.1; R Core Team, 2023; R Foundation for Statistical Computing, Vienna, Austria). A meta-analysis using a random intercept logistic regression model was conducted, and pooled outcome event rates and 95% confidence intervals (CIs) were calculated. The random effects model was used due to the expected high heterogeneity among the studies. Statistical heterogeneity was assessed by calculating I^2^ values. Inverse variance with logit transformation was used as a meta-analysis method. Odds ratios (ORs) and 95% CIs for outcome events between different medication regimens were calculated. The pooled outcome event rates were calculated using the ‘metaprop’ function from the ‘meta’ package version 5.1 − 1. Furthermore, to focus on outcome events 3 months after surgery, analysis was performed from studies reporting event rates for both 3 months and 1 year. The event rate for the time period from 3 months to 1 year was calculated by subtracting the 3-month event rate from the 1-year event rate. Publication bias was analysed by drawing funnel plots.

### Protocol registration

This review has been registered with PROSPERO (ID: CRD42024503612) and can be accessed via https://www.crd.york.ac.uk/prospero/display_record.php?RecordID=503612.

## Results

### Search results

**Fig. 1:**
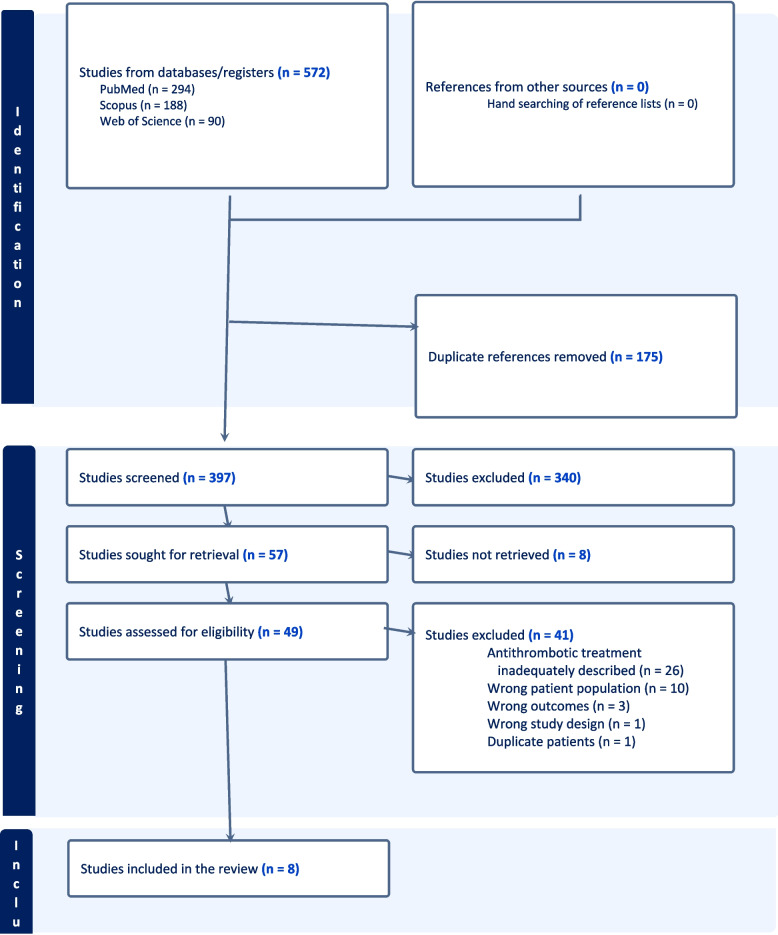
PRISMA flow chart for study screening

The literature search retrieved 572 abstracts. Since no eligible RCTs were identified in the initial search, the screening was expanded to cover suitable observational studies. After abstract screening, 49 full texts were assessed for eligibility, and eight observational studies were included (Fig. 1) [10, 19–25].

### Study and patient characteristics

Five of the studies were conducted in Europe (two in Germany and one each in Denmark, France and Switzerland) and three in North America (two in Canada and one in the USA) (Table 1). The study periods expanded through a 35-year time span from 1982 to 2017. The number of patients in the medication groups varied from 548 to 2384, resulting in a total of 6727 patients. The mean ages were between 55 and 77 years, and the proportion of female patients was 17–59%. Three of the studies also included patients with a history of atrial fibrillation, with the proportion of these patients being 5–43%. In these studies, the outcome event rates did not show a prominent increase when compared to the other studies. Five studies reported the rate of concomitant coronary artery bypass grafting surgery, and the rate varied from 0% to 36%.

**Table 1:** Study characteristics

**Study**	**Country**	**Study period**	**Mean age**	**Proportion of females (%)**	**Preoperative atrial fibrillation (%)**	**Concomitant coronary artery bypass surgery (%)**	**Aortic valve prosthesis used**	**Antithrombotic medication**	**Number of patients**	**Outcomes at 3 months after surgery**			**Outcomes at 1 year after surgery**		
										**Bleeding event, n (%)**	**Stroke, n (%)**	**Death, n (%)**	**Bleeding event, n (%)**	**Stroke, n (%)**	**Death, n (%)**
Gryaznov et al. (2020) [19]	USA	2013–2017	70	36%	43%	Not reported	Not specified	No antithrombotic medication	135	2 (1.5)	3 (2.2)	2 (1.5)	2 (1.5)	3 (2.2)	5 (3.7)
								Antiplatelet (ASA; combined with P2Y12 inhibitor in in 6.6%)	544	11 (2.0)	9 (1.7)	2 (0.4)	25 (4.6)	18 (3.3)	12 (2.2)
								Anticoagulant (warfarin in 90.6% or direct factor Xa inhibitor in 9.4%)	106	6 (5.7)	2 (1.9)	5 (4.7)	8 (7.5)	4 (3.8)	7 (6.6)
								Antiplatelet + anticoagulant (ASA in 97.5%; P2Y12 inhibitor in 2.5%; warfarin in 90%, direct factor Xa inhibitor in 10%)	326	14 (4.3)	10 (3.1)	4 (1.2)	27 (8.3)	13 (4.0)	15 (4.6)
Owais et al. (2016) [20]	Germany	2008–2015	72	59%	0	4%	Not specified	ASA	433	12 (2.8)		5 (1.2)			11 (2.5)
								3 months of warfarin followed by ASA	430	12 (2.8)		9 (2.1)			15 (3.5)
Mérie et al. (2012) [21]	Denmark	1997–2009	Not reported	Not reported	Not reported	Not reported	Not specified	Warfarin	2278			11 (0.5)			15 (0.7)
								Warfarin combined with ASA	916			5 (0.5)			10 (1.1)
								ASA	181			14 (7.7)			40 (22)
								No antithrombotic medication	700			47 (6.7)			98 (14)
Al-Atassi et al. (2012) [22]	Canada	Not defined	72	32%	0	21%	Carpentier-Edwards Perimount (86%), Medtronic Hancock II (11%), Medtronic Mosaic (3%)	Warfarin combined with ASA	28		0 (0)	0 (0)		0 (0)	0 (0)
							Carpentier-Edwards Perimount (79%), Medtronic Hancock II (18%), Medtronic Mosaic (3%)	ASA	28		0 (0)	0 (0)		0 (0)	0 (0)
Weber et al. (2012) [23]	Switzerland	2000–2009	55	17%	0	Not reported	Carpentier-Edwards 3000TFX and 3300TFX	ASA	103			5 (4.9)			10 (9.7)
Brueck et al. (2007) [10]	Germany	2001–2003	73	53%	0	0	Not specified	ASA	132	0 (0)	1 (0.8)	6 (4.5)	2 (1.5)	2 (1.5)	6 (4.5)
								No antithrombotic medication	156	0 (0)	2 (1.3)	8 (5.1)	1 (0.6)	2 (1.3)	8 (5.1)
Ninet et al. (1998) [24]	France	1982–1996	77	57%	10%	0	Sorin Group Mitroflow	ASA	113						10 (8.8)
Alex et al. (2018) [25]	Canada	1995–2014	62	29%	5%	36%	Carpentier–Edwards, Medtronic Mosaic, Medtronic Hancock II and MitroFlow valves, among others	3-month warfarin followed by ASA	118						4 (3.4)

### Risk of *bias*

The overall risk of bias was low in one, moderate in two, and serious in five of the included studies (Supplementary). Most issues were due to confounding factors and possible unrecognised deviations from the intended interventions.

### Mortality

**Fig. 2:**
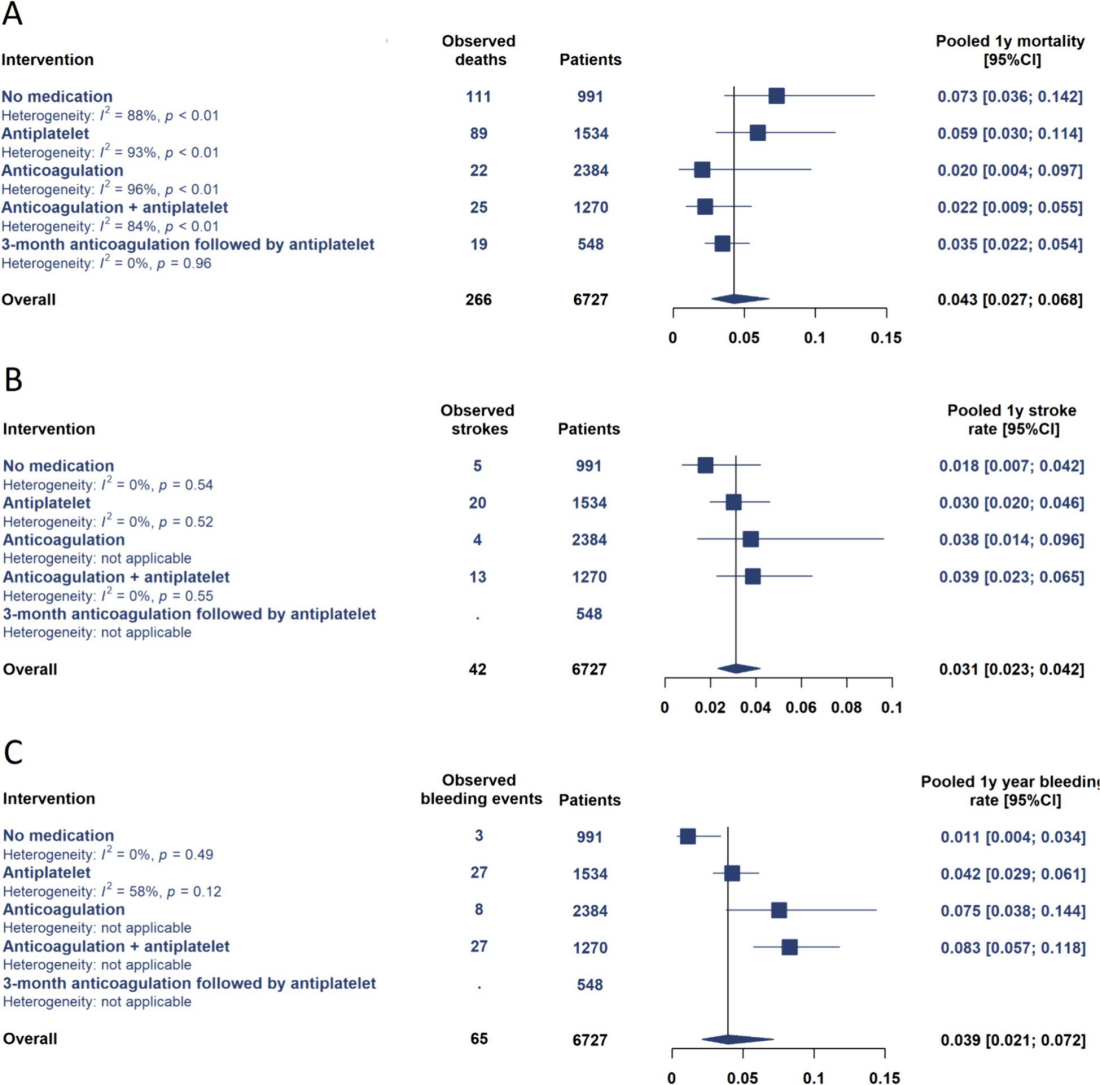
Pooled outcome event rates by antithrombotic treatment groups during 0–12 months after surgery. (**A**) mortality, (**B**) strokes, (**C**) bleeding events. CI = confidence interval

**Fig. 3:**
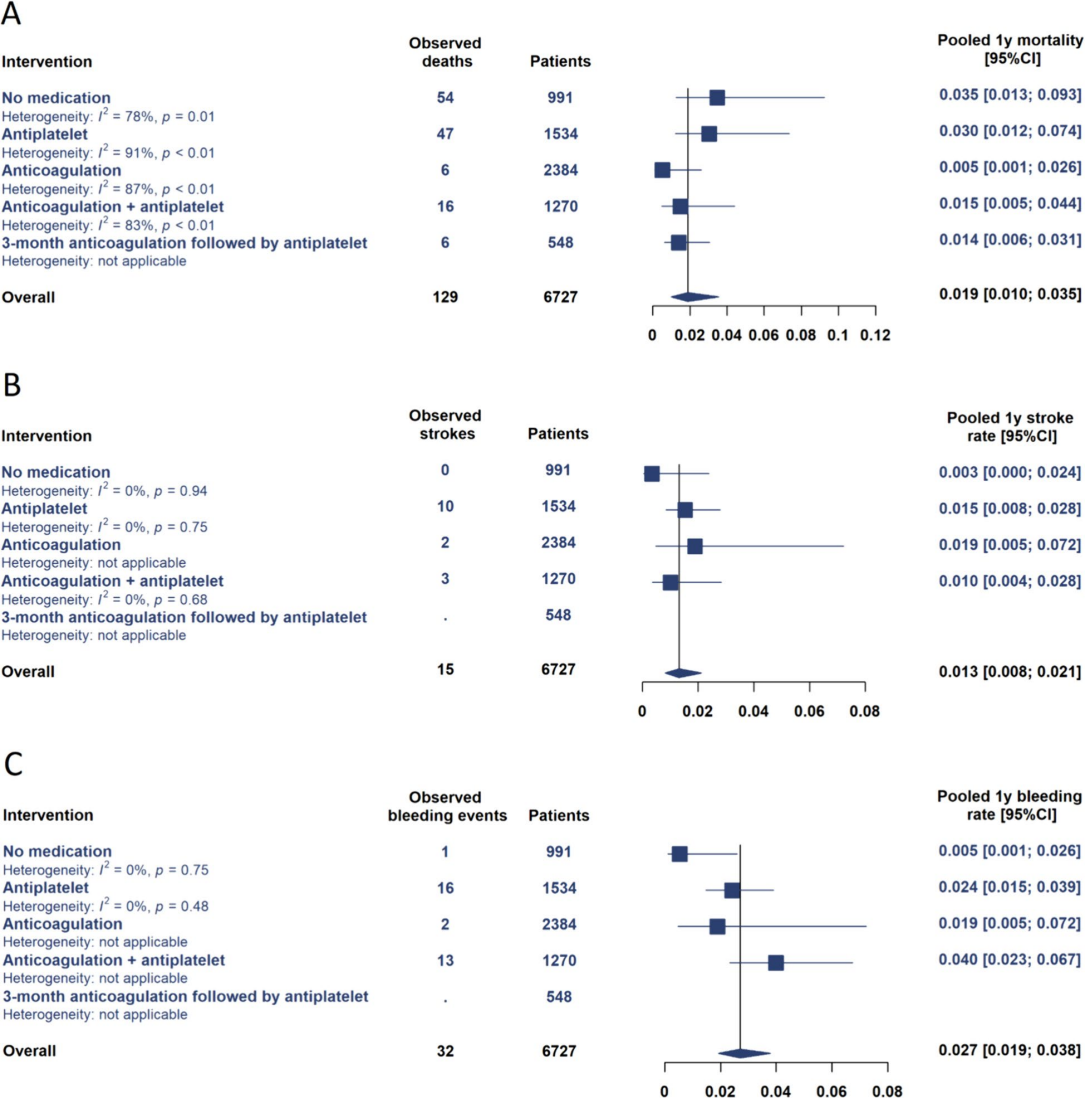
Pooled outcome event rates by antithrombotic treatment groups during 3–12 months after surgery. CI = confidence interval

Overall mortality during the first year after surgery was assessed in all the included studies (*N* = 6727 patients). Overall pooled mortality was 4.3% (95% CI 2.7–6.8%; Fig. 2). The lowest mortality rate was observed in patients with anticoagulation (2.0%, 95% CI 0.4–9.7%) and anticoagulation combined with antiplatelet therapy (2.2%, 95% CI 0.9–5.5%), and the highest was in patients without antithrombotic medication (7.3%, 95% CI 3.6–14.2%). Statistical heterogeneity, as shown by I^2^ values, was high, except in patients with 3-month anticoagulation followed by antiplatelet therapy. According to the funnel plot, there was a suspicion of publication bias, although the bias was balanced (Supplementary). The odds ratios for mortality did not show unequivocal evidence for the superiority of antithrombotic medications compared to no medications (Table 2). In the assessment of mortality from 3 months after surgery, the mortality was lower in the anticoagulant patients (0.5%, 95% CI 0.1–2.6%) than in antiplatelet patients (3.0%, 95% CI 1.2–7.4%) and those without antithrombotics (3.5%, 95% CI 1.3–9.3%; Fig. 3). However, the ORs of anticoagulation for mortality compared to the no medication group suggested only a trend towards a mortality benefit of anticoagulation. Evidence certainty was ranked as very low due to heterogeneity, risk of bias, imprecision and the low number of included studies per medication strategy.

**Table 2:** Odds ratios for outcome events of each antithrombotic medication, with patients without antithrombotic treatment set as a reference

	Mortality OR (95% CI)	Stroke OR (95% CI)	Bleeding OR (95% CI)
*0–12 months*			
Antiplatelet	0.85 (0.27–2.66)	1.71 (0.64–4.60)	3.90 (1.17–13.0)
Anticoagulation	0.27 (0.06–1.18)	2.16 (0.57–8.21)	7.21 (1.87–27.7)
Anticoagulation + antiplatelet	0.30 (0.07–1.25)	2.22 (0.78–6.26)	7.97 (2.39–26.6)
3-month anticoagulation followed by antiplatelet	0.48 (0.11–2.15)	NA^a^	NA^a^
*3–12 months*			
Antiplatelet	1.00 (0.23–4.32)	4.55 (0.58–35.6)	4.61 (0.86–24.7)
Anticoagulation	0.16 (0.02–1.01)	5.60 (0.50–62.4)	3.59 (0.43–30.2)
Anticoagulation + antiplatelet	0.46 (0.08–2.52)	2.96 (0.32–27.5)	7.76 (1.42–42.4)
3-month anticoagulation followed by antiplatelet	0.44 (0.05–3.94)	NA^a^	NA^a^

^a^Stroke or bleeding rates were not reported for patients with 3-month anticoagulation followed by antiplatelet

### Stroke

The stroke rate at 1 year after surgery was assessed in three studies (*N* = 1455 patients). The overall pooled stroke rate was 3.1% (95% CI 2.3−6.5%). During the 3–12 months after surgery, the stroke rate was 1.3% (95% CI 0.8–2.1%), with only 15 observed strokes across the studies. The funnel plots did not suggest the existence of publication bias, and heterogeneity was low. However, CIs were wide, and therefore, eligible evidence of differences was not observed between the antithrombotic treatment strategies at either 0- to 12-month or 3- to 12-month follow-up. Evidence certainty was ranked as very low due to risk of bias, imprecision and the low number of included studies per medication strategy.

### Bleeding

Bleeding rate was assessed in two of the included studies (*N* = 1399 patients). The overall pooled bleeding rate was 3.9% (95% CI 2.1–7.2%). There was, at most, a slight risk of publication bias, according to the funnel plots. At 0- to 12-month follow-up, all antithrombotic treatment regimens resulted in an increased bleeding rate compared to antithrombotic medication, with the highest bleeding rate identified in anticoagulation combined with antiplatelet patients. At 3- to 12-month follow-up, the findings were not as equivocal. The point estimates of antiplatelet and anticoagulation patients indicated an increased bleeding rate when compared to patients without medication, but the extremely wide CIs of ORs overlapped, indicating only a trending level difference between these groups. In patients with anticoagulation combined with antiplatelet therapy, there was up to an eight-fold increase in the bleeding rate when compared to patients with no antithrombotics, and this was also shown in the odds ratio (7.76, 95% CI 1.42–42.4). Evidence certainty was ranked as very low due to risk of bias, imprecision and the low number of included studies per medication strategy.

## Discussion

In the current meta-analysis, patients on anticoagulation and on anticoagulation combined with antiplatelet therapy showed the lowest mortality rates, while those without antithrombotic medication faced the highest mortality. However, the odds ratios for mortality did not decisively favour any antithrombotic regimen over no medication. After the initial 3 months, anticoagulation suggested a trending level potential mortality benefit compared to antiplatelet use and no medication. However, wide CIs and overlap with the no-medication group imply a tentative rather than conclusive mortality advantage for anticoagulation.

The anticoagulation group consisted mainly of patients with warfarin (99.6%). In this group, mortality was lowest during the 3- to 12-month period. Unexpectedly, the evidence of differences in stroke rates between the groups was uncertain, and the early bleeding rate was substantially higher in the anticoagulation group than in patients without antithrombotic medication. This suggests that although the rates of serious complications related to blood clotting and antithrombotic medication were not lower in patients with anticoagulation, anticoagulation may have a direct beneficial effect on mortality in relation to other medication regimens. It has been reported that the most common causes of death during the first year after SAVR are endocarditis, sepsis, heart failure and sudden cardiac death [26–28]. Furthermore, it has been reported that even up to one-quarter of SAVR patients will have myocardial microinfarctions during the 6 months following surgery and that the risk for infarct seems even higher in patients without preoperative coronary artery disease [29]. The resulting subclinical myocardial injury has been shown to increase the risk of sudden cardiac death [30]. It has been proposed that microinfarctions may be caused by microemboli dislodged from the valve prosthesis [31]. The findings of this meta-analysis encourage further investigation of these hypotheses. In addition, data on the outcomes of direct factor Xa inhibitors as an anticoagulative agent are needed, since among patients with bioprosthetic mitral valve, the outcomes have been shown to be non-inferior to warfarin [32].

In contrast, the outcomes in the antiplatelet group, consisting mainly of patients using ASA (97.7%), were unexpectedly unfavourable, as no evident benefit on mortality or stroke rate was observed, even when compared to patients without antithrombotic medication. However, the early bleeding rate seemed to be even higher. This is, at least to some extent, contradictory to prospective studies reporting equal outcomes with ASA and warfarin at 3–6 months after SAVR [33–35]. In this meta-analysis, 53% of deaths during the first year after SAVR in the antiplatelet group occurred after the first 3 months, whereas in the anticoagulation group, the respective proportion was 27%, suggesting that after the early postoperative period, mortality remains higher without proper anticoagulation. These findings suggest that ASA as monotherapy may not be an advisable antithrombotic treatment strategy after SAVR using a biological prosthesis.

With regard to stroke rates, wide CIs precluded the observation of eligible evidence for differences between antithrombotic treatment strategies at 0–12 and 3–12 months after surgery. Surprisingly, the point estimate of the stroke rate was lowest in the no antithrombotic medication group, which may be seen as controversial in light of the intended effect of antithrombotic medications in preventing prosthesis valve thrombosis formation and thereby preventing stroke. The stroke rate in patients with no antithrombotic medication was derived from the results of a single study by Gryaznov et al. (2020), in which there was a 43% rate of atrial fibrillation history prior to surgery and thereby an indication for anticoagulation even before surgery [19]. The low stroke rate in patients without antithrombotic medication may reflect a lower risk of haemorrhagic stroke [36, 37]. When comparing the haemorrhagic stroke rates, which were reported by Gryaznov et al. (2020) separately from ischemic strokes, no significant differences were observed between the treatment groups, with a rate of less than 1% in all treatment groups [19]. Thus, there might have been other underlying comorbidities predisposing to stroke rather than the effect of antithrombotic medication falling short of expectations.

At 0- to 12-month follow-up, higher bleeding rates were observed in all antithrombotic regimens compared to those without medication, peaking in anticoagulation combined with antiplatelet therapy. At 3- to 12-month follow-up, the findings were less definitive, with trends of increased bleeding in the antiplatelet and anticoagulation groups, but the wide CIs suggested only a potential difference. However, patients on anticoagulation combined with antiplatelet therapy exhibited up to an eight-fold increase in bleeding rates compared to those without antithrombotics.

Overall, the evidence certainty was very low, which can be attributed to methodological factors and retrospective data. Along with uncontrolled confounding and implicit multidimensionality behind thromboembolic events after biological SAVR, the results of individual studies held a considerable risk of bias. These factors inevitably affected the results and their interpretation in this meta-analysis. With these considerations in mind, the future implications of this meta-analysis include the following. First, anticoagulation therapy using warfarin showed a tendency towards a benefit in mortality at 1 year after surgery when compared to antiplatelet therapy or no antithrombotic medication patients. Second, anticoagulation combined with antiplatelet therapy, antiplatelet therapy with ASA alone and no antithrombotic medication resulted in inferior overall outcomes. Therefore, according to this meta-analysis of observational studies, these antithrombotic medication strategies may not be advisable after SAVR using a biological prosthesis. Third, there seemed to be a higher rate of bleeding events among anticoagulant patients than among antiplatelet patients and no antithrombotic medication patients during the 0- to 12-month follow-up, but at the 3- to 12-month follow-up, the difference was not as clear. This suggests a higher bleeding rate in anticoagulation patients during the first 3 months after surgery, after which the rate seems to even out.

In summary, the results of this meta-analysis cautiously suggest that continuing with anticoagulative medication as the antithrombotic medication strategy may be beneficial 3 months after SAVR using a biological prosthesis. However, attributable to methodological factors, the very low certainty of evidence emphasises the need for cautious interpretation and underscores the imperative for more robust research using randomised data to clarify optimal antithrombotic strategies in this patient population. In addition, there is a lack of data on direct factor Xa inhibitors and P2Y_12_ inhibitors as antithrombotic medication strategies 3 months after surgery.

### Limitations

The absence of RCT data limits the credibility of our findings, as this analysis relies solely on observational studies, including plausible selection bias and potential inaccuracies and deviations from the reported antithrombotic medication regimens. Additionally, the lack or scarcity of information regarding concomitant procedures and comorbidities (especially cerebrovascular disease, separate rates of ischemic and haemorrhagic strokes and arrhythmias, such as atrial fibrillation) and follow-up data on medication usage in the included studies increases uncertainty and adds evidently uncontrolled confounding to our results. Moreover, the variation in prosthesis models and their haematogenic properties among the studies introduces potential heterogeneity. Patient groups, such as paediatric and pregnant patients as well as renal insufficiency patients requiring dialysis, were excluded to avoid special considerations related to antithrombotic treatment in these patient groups. In addition, we did not include TAVI patients; therefore, our results are not generalisable in these patients. These limitations underscore the need for cautious interpretation and highlight areas for future research to enhance our understanding of optimal antithrombotic management in this patient population. A well-designed and appropriately powered RCT is warranted.

## Conclusion

In conclusion, despite revealing a tendency towards the benefit of anticoagulation therapy in terms of mortality at 1 year after biological SAVR and suggesting a potential advantage in continuing anticoagulation beyond 3 months, this meta-analysis is limited by very low evidence certainty. The imperative for cautious interpretation and the urgent need for more robust randomised research underscore the complexity of determining optimal antithrombotic strategies in this patient population.

### Supplementary Information

Additional file 1: Supplementary 1.Risk of bias assessment for seven domains and overall for the included studies according to the ROBINS-I (2016) tool. Supplementary 2. Funnel plots showing possible publication bias by antithrombotic treatment groups 0–12 months after surgery. A: mortality, B: strokes, C: bleeding events. Supplementary 3. Funnel plots showing possible publication bias by antithrombotic treatment groups 3–12 months after surgery. A: mortality, B: strokes, C: bleeding events. Supplementary 4. Mortality rates in antithrombotic treatment groups 0–12 months after surgery for each included study. CI = confidence interval. Supplementary 5. Mortality rates in antithrombotic treatment groups 3–12 months after surgery for each included study. CI = confidence interval. Supplementary 6. Stroke rates in antithrombotic treatment groups 0–12 months after surgery for each included study. CI = confidence interval. Supplementary 7. Stroke rates in antithrombotic treatment groups 3–12 months after surgery for each included study. CI = confidence interval. Supplementary 8. Bleeding rates in antithrombotic treatment groups 0–12 months after surgery for each included study. CI = confidence interval. Supplementary 9. Bleeding rates in antithrombotic treatment groups 3–12 months after surgery for each included study. CI = confidence interval.

## Data Availability

The data supporting the results of our analysis is provided within the manuscript in Table 1.

## Abbreviations

AHA: American Heart Association
ASA: acetylsalicylic acid
CI: Confidence interval
ESC: European Society of Cardiology
GRADE: Grading of Recommendations Assessment, Development and Evaluation framework
MOOSE: Meta-analysis of Observational Studies in Epidemiology checklist
OR: Odds ratio
PRISMA: Preferred Reporting Items for Systematic Reviews and Meta-Analysis guidelines
RCT: Randomised controlled trial
ROBINS-I: The Risk of Bias in Non-randomized Studies – of Interventions assessment tool
SAVR: surgical aortic valve replacement
TAVI: transcatheter aortic valve implantation
