# Supplementary material for: Antithrombotic management after aortic valve replacement with biological prosthesis: a meta-analysis
Source: J Cardiothorac Surg. 2024 Jun 26;19:385. doi: 10.1186/s13019-024-02863-z (PMC11202358; doi:10.1186/s13019-024-02863-z)
Supplement: Supplementary file 1 — Additional file 1: Supplementary 1.Risk of bias assessment for seven domains and overall for the included studies according to the ROBINS-I (2016) tool. Supplementary 2. Funnel plots showing possible publication bias by antithrombotic treatment groups 0–12 months after surgery. A: mortality, B: strokes, C: bleeding events. Supplementary 3. Funnel plots showing possible publication bias by antithrombotic treatment groups 3–12 months after surgery. A: mortality, B: strokes, C: bleeding events. Supplementary 4. Mortality rates in antithrombotic treatment groups 0–12 months after surgery for each included study. CI = confidence interval. Supplementary 5. Mortality rates in antithrombotic treatment groups 3–12 months after surgery for each included study. CI = confidence interval. Supplementary 6. Stroke rates in antithrombotic treatment groups 0–12 months after surgery for each included study. CI = confidence interval. Supplementary 7. Stroke rates in antithrombotic treatment groups 3–12 months after surgery for each included study. CI = confidence interval. Supplementary 8. Bleeding rates in antithrombotic treatment groups 0–12 months after surgery for each included study. CI = confidence interval. Supplementary 9. Bleeding rates in antithrombotic treatment groups 3–12 months after surgery for each included study. CI = confidence interval. [file 13019_2024_2863_MOESM1_ESM.docx]

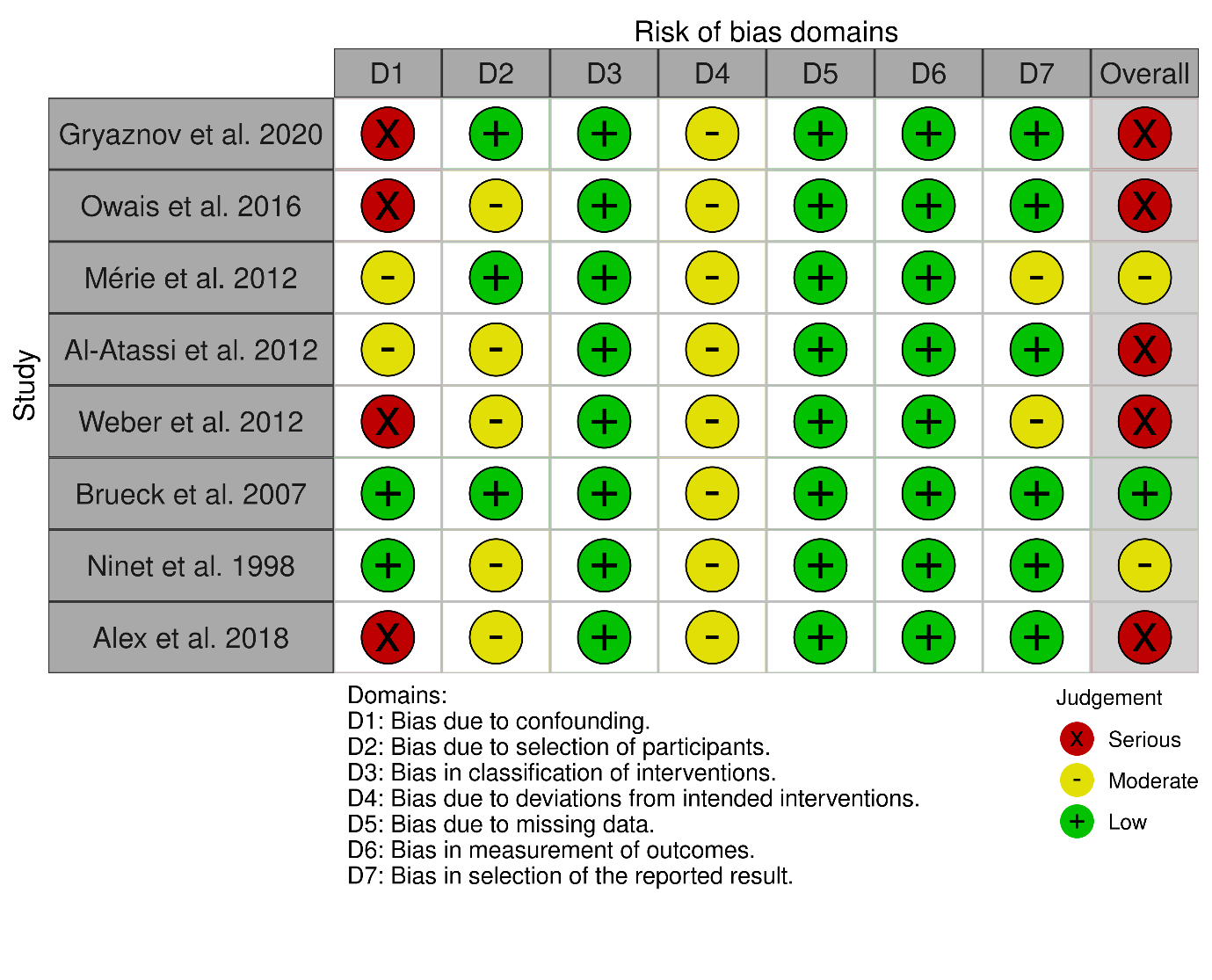


**Supplementary 1.** Risk of bias assessment for seven domains and overall for the included studies according to the ROBINS-I (2016) tool.


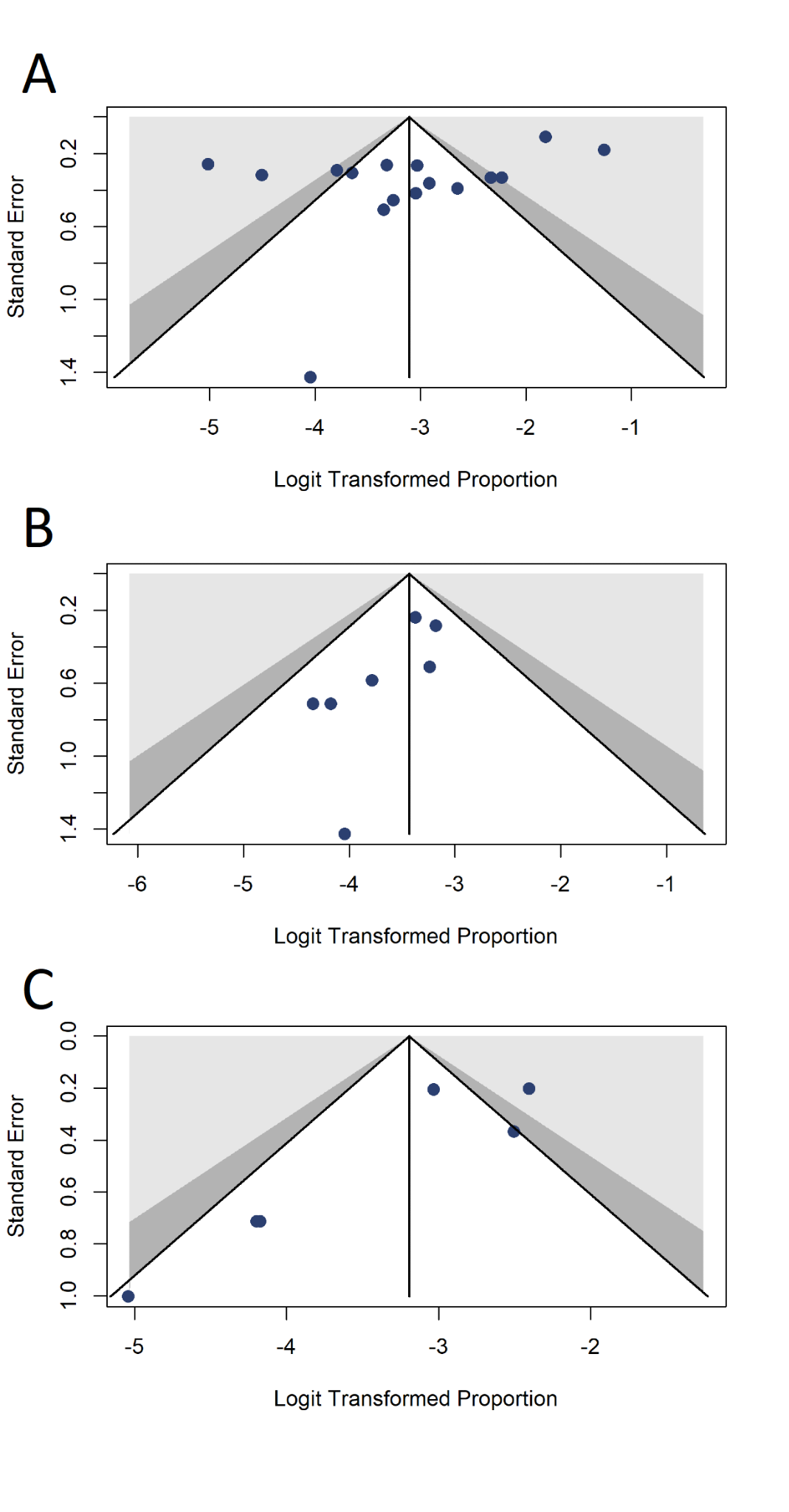


**Supplementary 2.** Funnel plots showing possible publication bias by antithrombotic treatment groups 0–12 months after surgery. A: mortality, B: strokes, C: bleeding events.


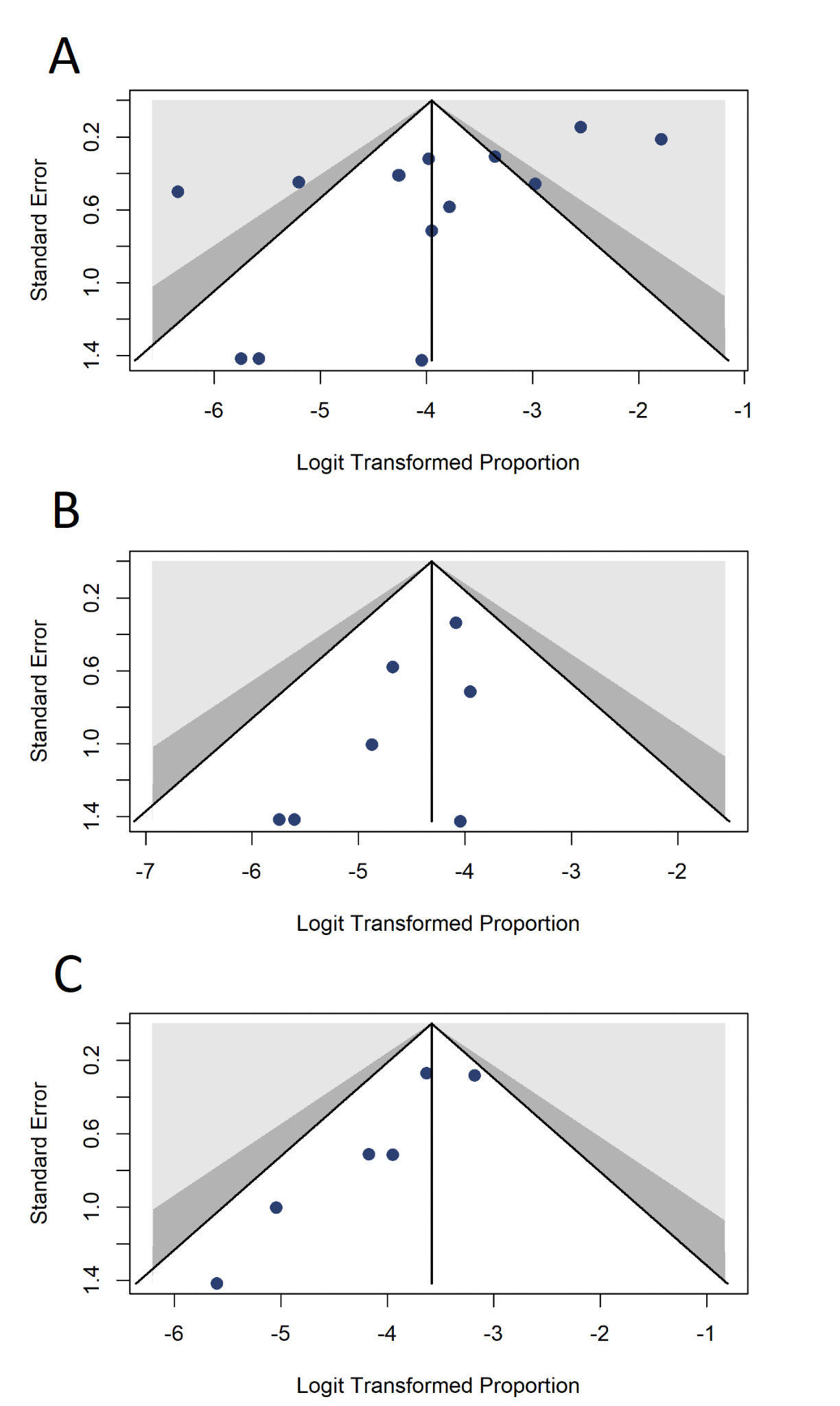


**Supplementary 3.** Funnel plots showing possible publication bias by antithrombotic treatment groups 3–12 months after surgery. A: mortality, B: strokes, C: bleeding events.


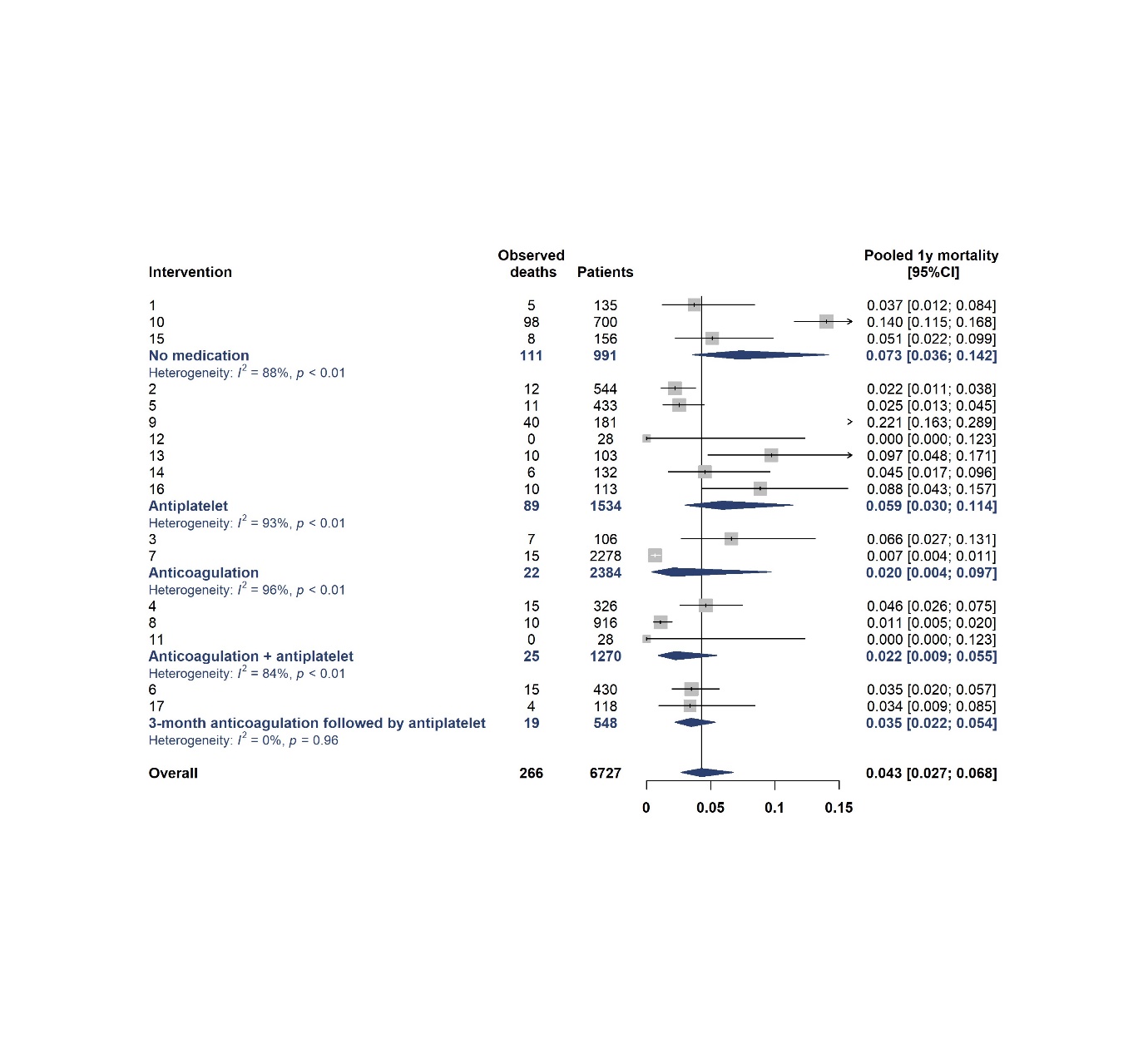


**Supplementary 4.** Mortality rates in antithrombotic treatment groups 0–12 months after surgery for each included study. CI = confidence interval.


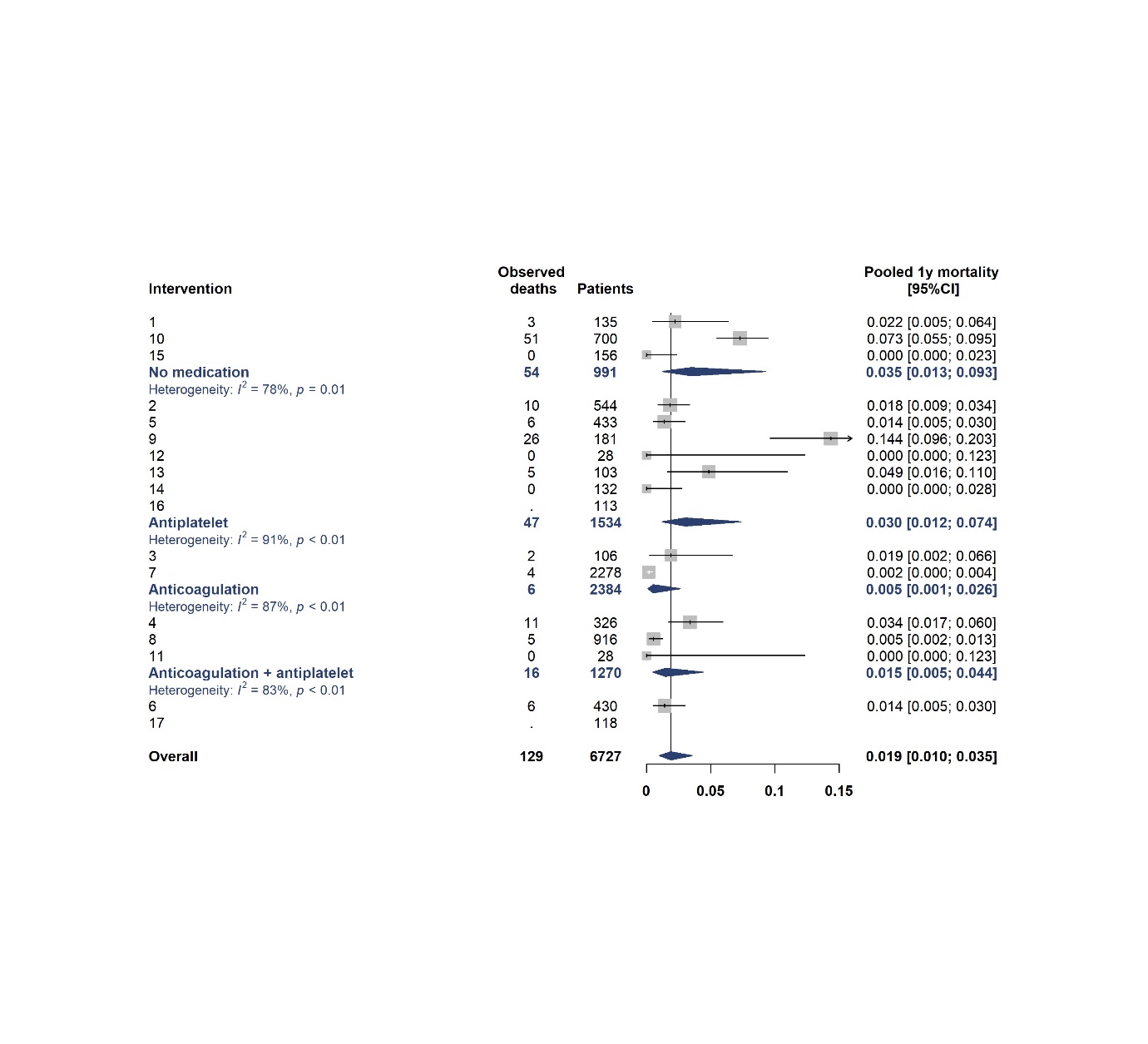


**Supplementary 5.** Mortality rates in antithrombotic treatment groups 3–12 months after surgery for each included study. CI = confidence interval.


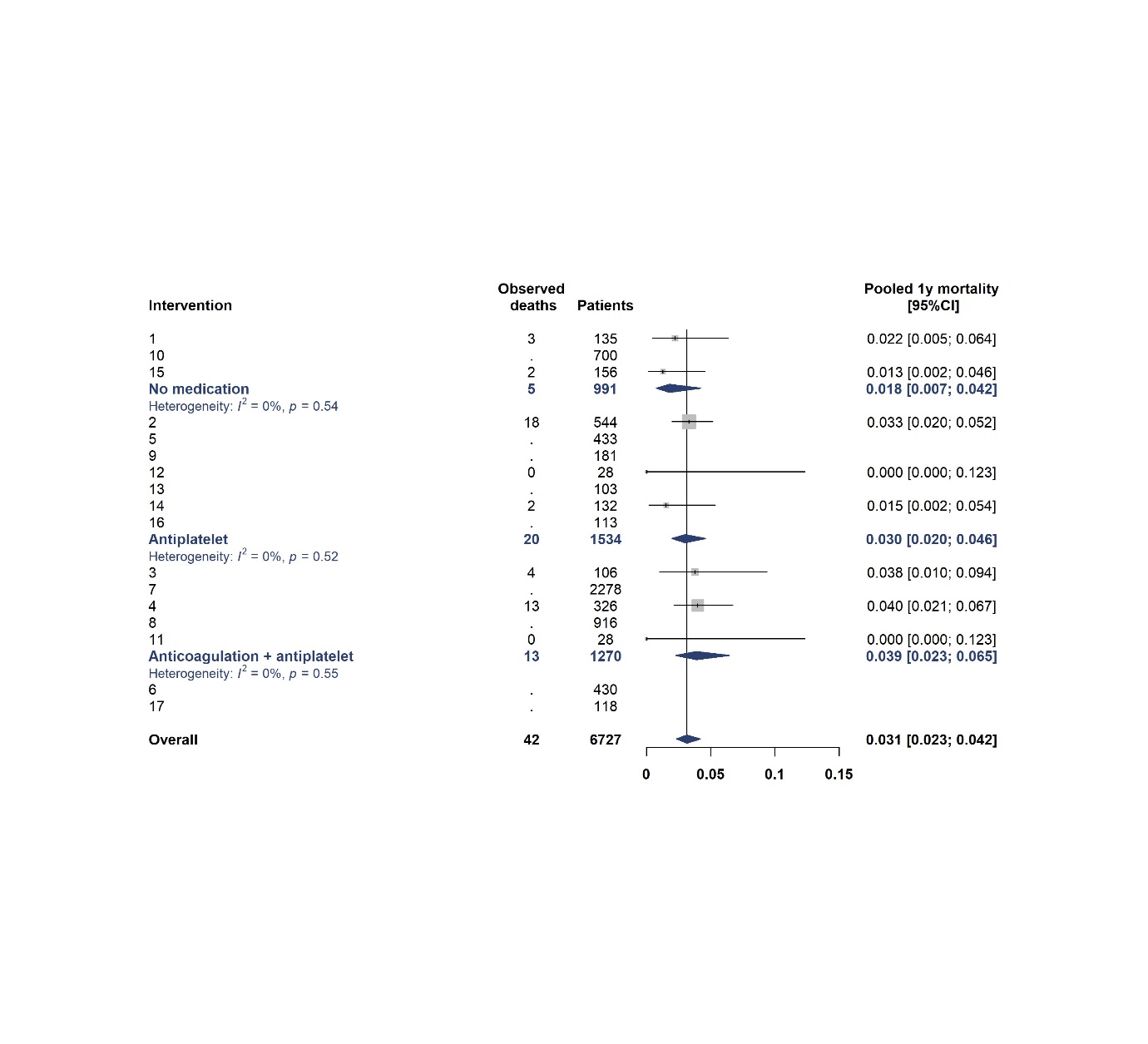


**Supplementary 6.** Stroke rates in antithrombotic treatment groups 0–12 months after surgery for each included study. CI = confidence interval.


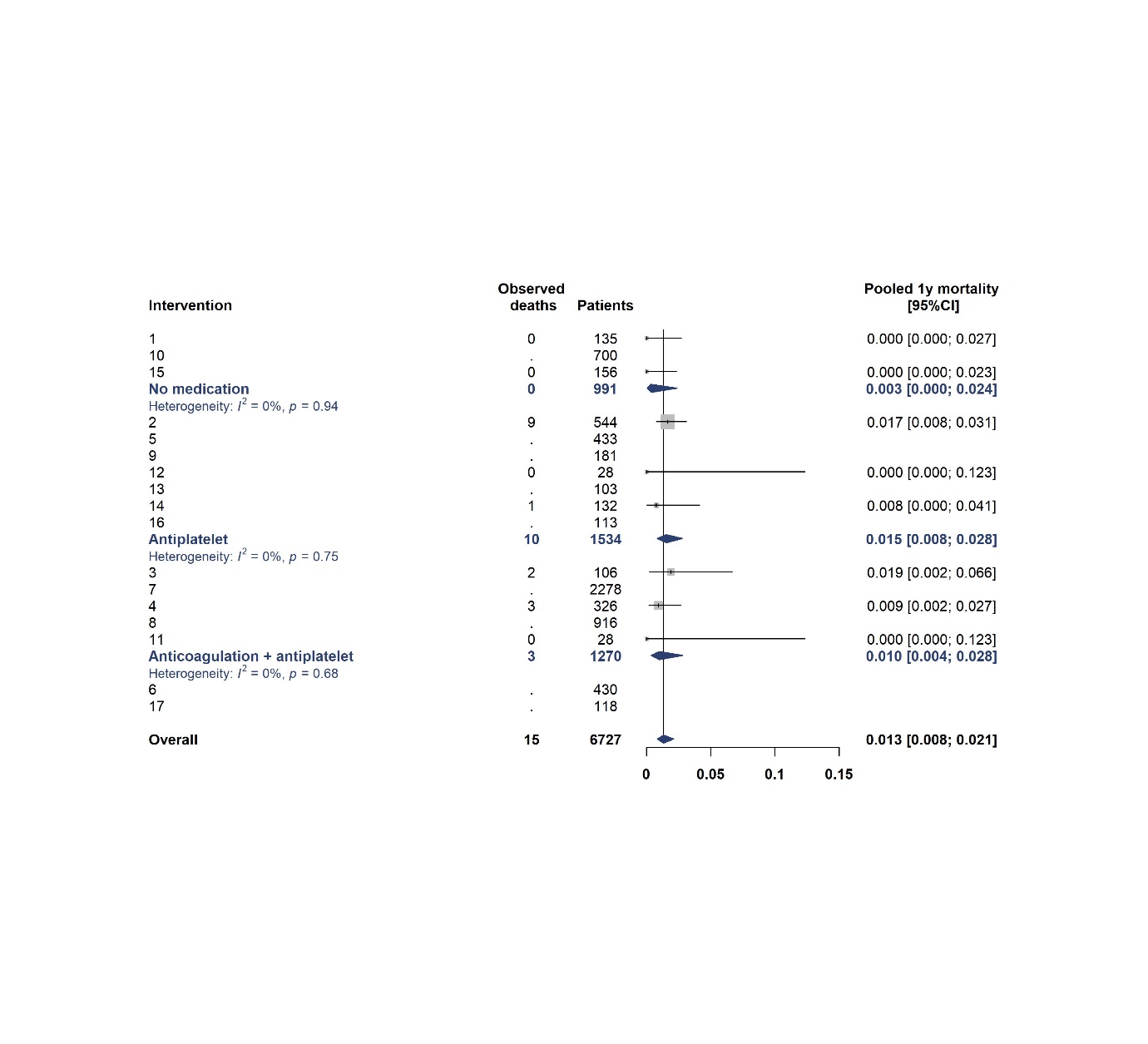


**Supplementary 7.** Stroke rates in antithrombotic treatment groups 3–12 months after surgery for each included study. CI = confidence interval.


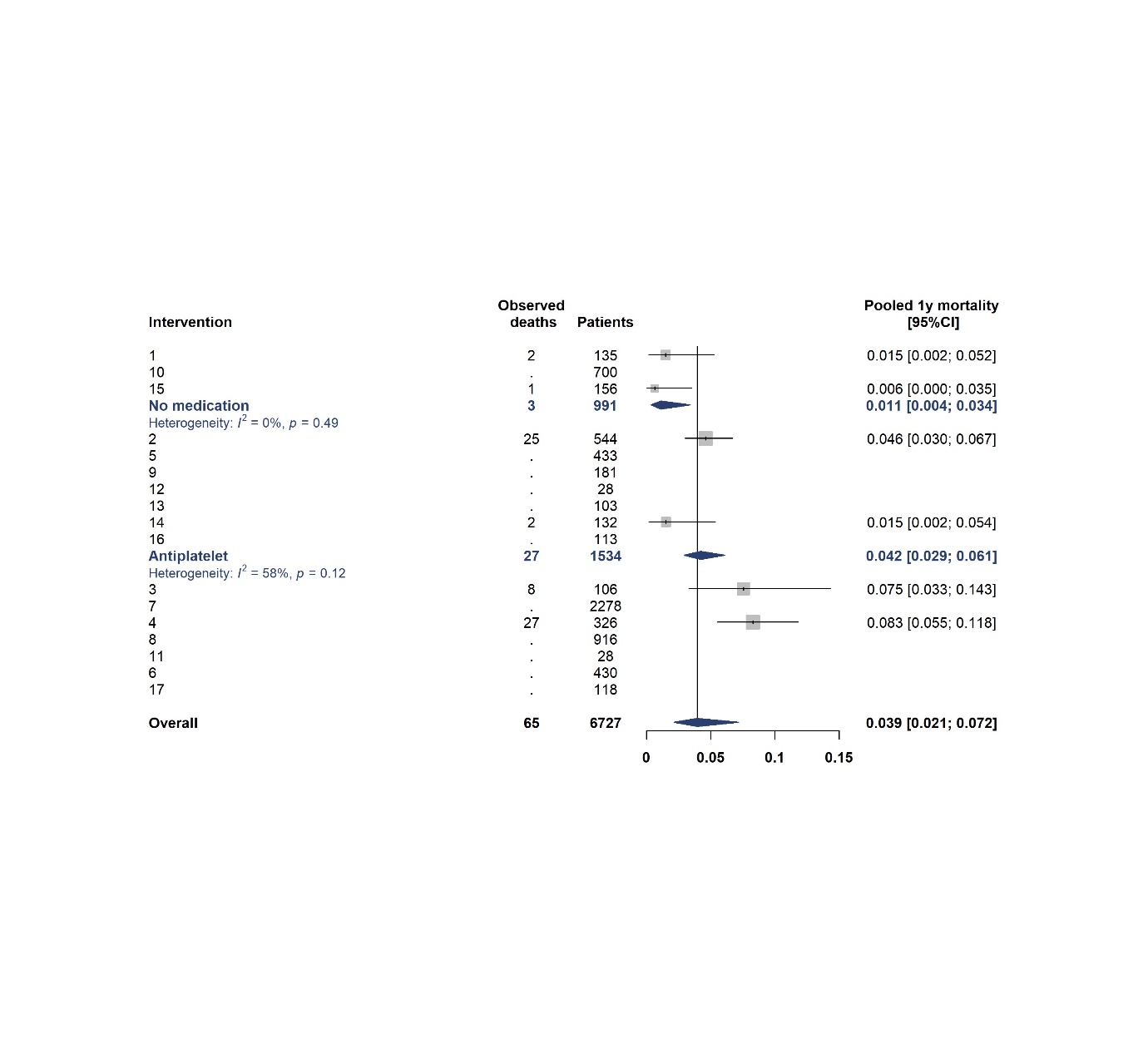


**Supplementary 8.** Bleeding rates in antithrombotic treatment groups 0–12 months after surgery for each included study. CI = confidence interval.


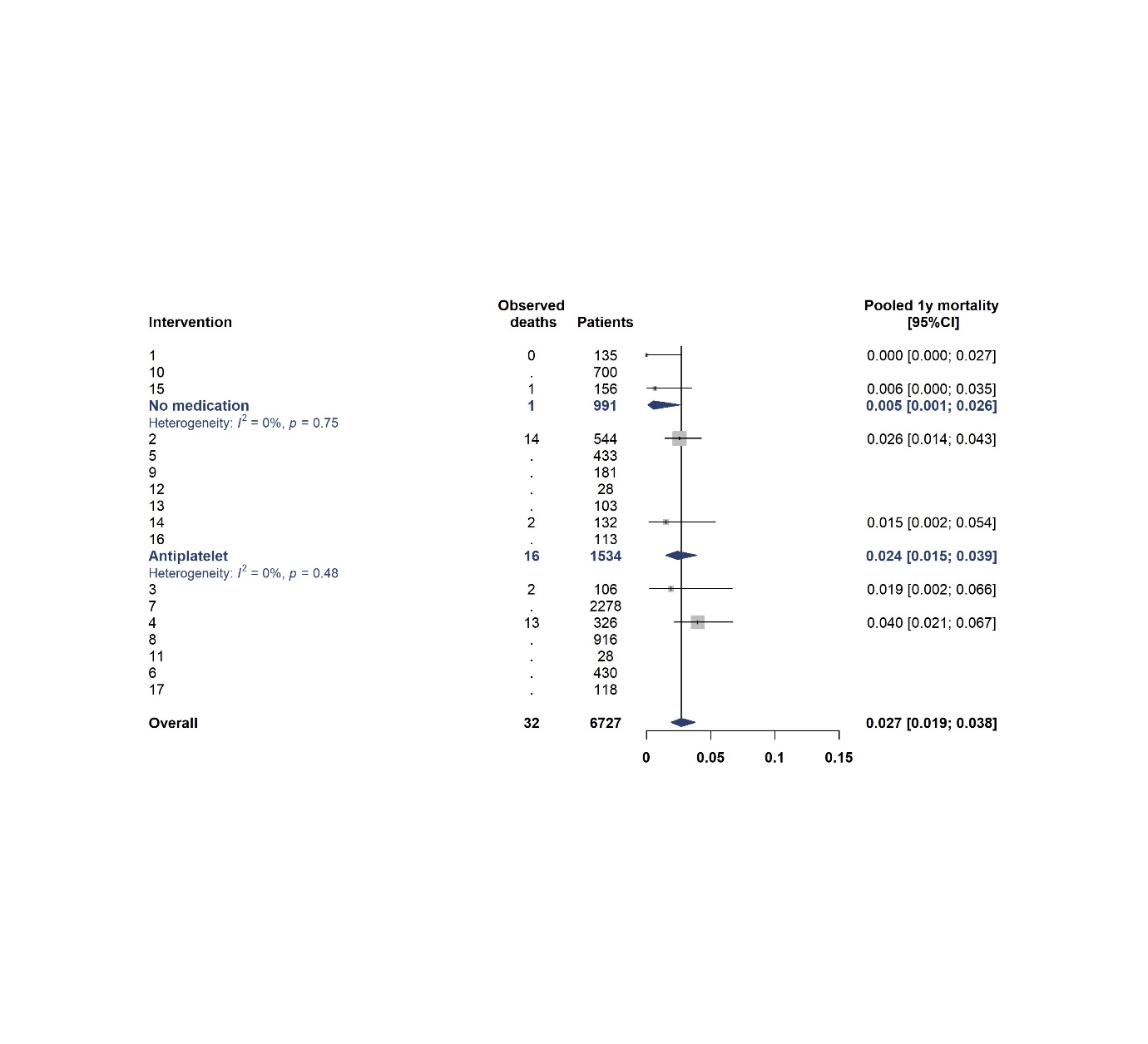


**Supplementary 9.** Bleeding rates in antithrombotic treatment groups 3–12 months after surgery for each included study. CI = confidence interval.
